# Neural correlates of conditioned pain responses in fibromyalgia subjects indicate preferential formation of new pain associations rather than extinction of irrelevant ones

**DOI:** 10.1097/j.pain.0000000000001907

**Published:** 2020-05-04

**Authors:** Angelica Sandström, Isabel Ellerbrock, Jeanette Tour, Diana Kadetoff, Karin Birgitta Jensen, Eva Kosek

**Affiliations:** a Department of Clinical Neuroscience, Karolinska Institutet, Stockholm, Sweden; b Department of Neuroradiology, Karolinska University Hospital, Stockholm, Sweden; c Stockholm Spine Center, Löwenströmska Hospital, Upplands Väsby, Sweden

**Keywords:** fMRI, Conditioned pain responses, Pain catastrophizing, Fibromyalgia, Healthy controls

## Abstract

Behavioral studies have demonstrated aberrant safety processing in fibromyalgia subjects (FMSs) and suggested that patients accumulate new potential pain-related threats more effectively than extinguishing no longer relevant ones. The aim of the current study was to investigate the neural correlates of conditioned pain responses and their relationship with emotional distress in FMS (n = 67) and healthy controls (HCs, n = 34). Using functional magnetic resonance imaging, we traced conditioned pain responses to an identical moderately painful pressure (P30) depending on whether it was following a green (P30green) or a red (P30red) cue. The cues were previously associated with individually calibrated painful pressure stimuli of low and high intensity, corresponding to visual analogue scale 10 and 50 mm, respectively. Fibromyalgia subjects displayed increased P30green ratings over time, while P30red ratings remained elevated. Healthy controls adapted all pain ratings to resemble moderate pain. Fibromyalgia subjects exhibited increased activation for [P30green>P30red] in M1/anterior insula, whereas HC showed increased S2/mid-insula response to [P30red>P30green]. High pain catastrophizing scale (PCS) ratings in fibromyalgia (FM) covaried with heightened brain activation for [P30green] × PCS in left dorsolateral prefrontal cortex and medial prefrontal cortex/orbitofrontal cortex; and [P30green>P30red] × PCS in dorsal anterior cingulate cortex/mid-cingulate cortex; superior temporal pole, extending to anterior insula; bilateral thalamus; and posterior insula. Psychophysiological interaction analysis for FM [P30green>P30red] × PCS revealed a dissociation in functional connectivity between thalamus and bilateral inferior parietal lobe. In alignment with behavioral data, FMS displayed a cerebral response suggesting preferential formation of new pain-related associations while simultaneously maintaining no longer relevant ones. The opposite was observed in HC. Increased responses to pain-related threats in FM may contribute to dysfunctional pain-protective behaviors and disability.

## Abstract 2

Supplemental Digital Content is Available in the Text.

Functional magnetic resonance imaging pain conditioning data suggest that fibromyalgia prioritizes updating their cerebral representation to forming new potential pain-related associations while simultaneously maintaining no longer relevant ones.

## 1. Introduction

Fibromyalgia (FM) is a chronic widespread musculoskeletal pain condition, a prototypical nociplastic disorder, associated with functional and structural changes in the central nervous system,^24^ such as dysfunctional descending pain modulation,^19,25,26^ aberrant opioid signaling,^38^ and increased cerebral glial activation.^2^ Although the neural processes of applied experimental pain have been widely investigated in fibromyalgia subjects (FMSs),^19,23,35,38^ it remains unclear to what extent emotional distress and dysfunctional emotional learning are related to chronic pain, and how these may alter neural circuits.^33^ Accumulating research suggest that pain in FM is influenced by notably complex cognitive processes, particularly related to perception of an increased threat of pain.^15,30–32,44^

The fear-avoidance model of pain assumes a vicious circle, in which the experience of pain (possibly caused by an injury) is interpreted as threatening (eg, through pain catastrophizing) and consequently develops into pain-related fear and excessive protective behaviors that may preserve pain disability.^43,44^ Within the framework of pain, conditioning takes place when a cue (conditioned stimulus [CS]) is repeatedly associated with a noxious stimulation or painful experience (unconditioned stimulus [US]), until the cue becomes pain-predictive and evokes a pain-related response by itself (conditioned response), eg, fear of pain. This sort of learning can be established with and without conscious awareness^16–18^ and takes place rapidly, to facilitate early and effective protection against injury and other bodily threats.^44^

Behavioral studies have demonstrated contingency learning deficits in FMS compared with other chronic pain patients and healthy controls (HCs).^15,30,31^ Specifically, FMS displayed impaired safety learning and excessive generalization of pain-related fear when exposed to cues that signal safety.^15,30,31^ Once pain-related fear has been established in FMS, it is difficult to extinguish.^32^ Collectively, these dysfunctional pain behaviors are hypothesized to lead to increased anxiety as an increased range of stimuli becomes capable of predicting harm.^15,30–32,44^ In FMS, catastrophizing has been shown to be associated with pain sensitivity and pain severity in studies using a pain paradigm where subjects were able to anticipate forthcoming painful stimulation,^8,14,41^ whereas studies using a pseudorandomized paradigm of applied painful pressure found no such association.^21^

To the best of our knowledge, the current study was the first to investigate the neural correlates of conditioned pain responses (not to be confused with conditioned pain modulation [CPM]) in FMSs vs HCs, and its relationship with emotional distress (ie, anxiety, depression, and catastrophizing) in FMS. Based on previous studies,^14,15,31^ we hypothesized that FMS would (1) exhibit a quicker increase in P30green ratings and slower decrement in P30red ratings in the test phase due to impaired safety learning and an attentional bias towards actual or potential pain-related threats. We expected these behavior patterns to be associated with (2) higher pain-related brain activation in regions associated with anticipation and attention, which in turn would be related to (3) increased ratings of emotional distress in FMS. Investigating factors that influence pain modulation is important for understanding chronic pain maintenance and developing effective therapies.

## 2. Method

### 2.1. Inclusion/exclusion criteria

Fibromyalgia subjects and HCs were recruited through advertisement in the daily press. All FMSs completed a physical examination by a specialist in rehabilitation medicine and pain relief (D.K.) on a separate occasion to ensure that they fulfilled the inclusion criteria. Inclusion criteria for FMSs were female sex, working age (20-60 years), right handed, and meet the ACR-1990 as well as the ACR-2011 classification criteria for FM.^46,47^ Exclusion criteria were other dominant pain conditions than FM, rheumatic or autoimmune diseases, other severe somatic diseases (neurological, cardiovascular, cancer, etc.), psychiatric disorders including ongoing treatment for depression or anxiety, substance abuse, pregnancy, magnetic implants, previous brain or heart surgery, hypertension (>160/90 mm Hg), obesity (body mass index > 35), smoking (>5 cigarettes/day), medication with antidepressants or anticonvulsants, inability to speak or understand Swedish, self-reported claustrophobia, not being able to refrain from non-steroidal anti-inflammatory drugs, analgesics, or hypnotics for at least 48 hours before study participation (48 hours before the first visit, and 72 hours before the second visit, ie, the scanning session). Healthy controls were right-handed women, age balanced to the FMSs, free from chronic pain and without regular medications with non-steroidal anti-inflammatory drugs, analgesics or sleep medication, and free from the exclusion criteria above. The HCs were screened by telephone.

Eighty subjects diagnosed with fibromyalgia and 40 sex- and age-balanced HCs were included in the current study. One FM and one HC were excluded due to drop out or incomplete behavioral data collection. Thus, behavioral data were analyzed from 79 FMSs patients and 39 HCs.

In total, functional MR data were analyzed from 67 FMSs and 34 HCs. Six FMSs and 2 HCs were excluded due to excessive head motion. One FMS was excluded because she reported closing her eyes during scan (thus unable to see the cue representations). One FMS was excluded due to structural brain anomalies. Four FMSs and 3 HCs were drop outs from the functional magnetic resonance imaging (fMRI) session or excluded due to technical issues.

### 2.2. Procedure

The current study forms the first part of a larger project (see study plan https://osf.io/8zqak). Subjects visited the laboratory on 2 subsequent days and completed one behavioral experimental session and one neuroimaging session. During the first day, the subjects completed questionnaires, provided saliva samples for genotyping, followed by the acquisition phase outside of the scanner (see below), assessment of pressure pain thresholds (PPTs), and a test of CPM. The data regarding the associations between pain-relevant functional genotypes and PPT, CPM, and neuroimaging data will be presented in upcoming articles. All subjects underwent the 3 phases of the conditioning experiment, ie, (1) an acquisition phase (CS is repeatedly associated with US) outside the scanner, (2) an acquisition phase (CS is repeatedly associated with US) inside the scanner, followed by (3) an experimental test phase (testing the strength of the conditioned response) inside the scanner. Only the results from the experimental test phase were analyzed in the current study. On the first day, all subjects filled out questionnaires concerning pain, depression, anxiety, pain catastrophizing, and other health-related quality of life measures (*see* Questionnaires). A pain sensitivity examination followed, including individual calibration of pressure pain sensitivity corresponding to a subjective rating of 10/100 (P10) and 50/100 (P50) mm using a plastic visual analogue scale (VAS, ranging from 0 mm = “no pain” to 100 mm = “worst imaginable pain”). Subjects moved a red plastic vertical line to rate their pain sensation following each painful pressure. Painful stimulation was achieved through applying cuff pain algometry (CPA, Hokanson E20/AG101) on the left calf (size 13 × 85 cm). The CPA device was chosen over other common methods of applying pain (eg, cutaneous heat on skin), as CPA may have a preferential effect on deep tissue nociceptors, which may be more clinically relevant for FM. The CPA has been successfully used in previous FM studies.^27^ Subjects were seated upright on a hospital stretcher with their legs extended in front of them. In order for the inflatable cuff to not touch the stretcher, one cushion was placed under the subjects' ankle and one cushion was placed under subjects' knee. During the subjective calibration of pain, participants received a manually initiated ascending series of 5-second stimuli with increasing steps of 25 mm Hg to determine the PPT (first VAS > 0 mm) and stimulation maximum (first VAS > 60 mm, max 424 mm Hg). Subjects rated their pain intensity on a plastic hand-held VAS scale. The PPT and stimulation maximum were followed by 2 randomized series of 5 stimuli to determine each subject's representation of VAS 10 mm (P10) and VAS 50 mm (P50). The randomized series to determine P10 used the PPT as a starting point and −2 steps and +2 steps of 25 mm Hg. The randomized series to determine P50 used the stimulation maximum as a starting point and −4 steps of 25 mm Hg. If the first subjective rating of 10-mm VAS was <100 mm Hg, increasing steps of 10 mm Hg were used for the randomized series determining P10 (instead of 25 mm Hg). Next, subjects completed an acquisition phase of our conditioning paradigm in the behavioral testing room (outside the scanner). During this phase, subjects were verbally instructed and trained in front of a computer monitor to associate a green circle with low pressure (their individually calibrated P10 stimulation [P10green]) and a red circle with high pressure (their individually calibrated P50 stimulation [P50red]), presented in a pseudorandomized order (10 × P10green; 10 × P50red). Visual cues were presented for 2 seconds, followed by a jittered anticipation phase, followed by 5-second pressure stimulation. After each stimulus, subjects rated their perceived pain on a computerized 100-mm VAS.

**Figure 1.**
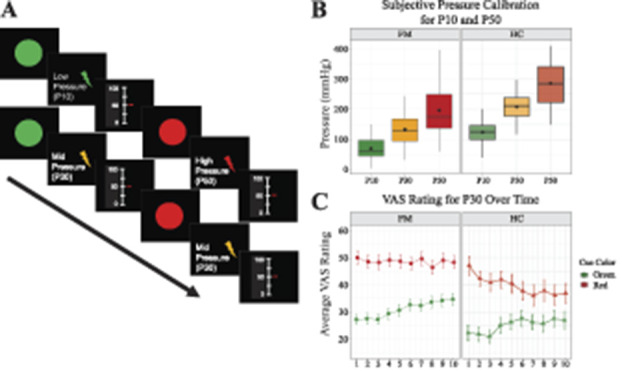
(A) Illustrates the experimental paradigm. Top row: exemplifies the conditioning paradigm in which green and red cues were followed by low and high painful pressure, respectively. Bottom row: exemplifies the experimental paradigm investigating conditioned pain responses in FM and HC. Here, both green and red cues were followed by an identical mid-intensity painful pressure. (B) Boxplots illustrate average pressure (mm Hg) corresponding to subjectively calibrated pain ratings of 10-mm VAS (P10) and 50-mm VAS (P50). P30 corresponds to each subjects' calculated average between P10 and P50. Fibromyalgia values are displayed in the plot to the left, and HC values are displayed in the plot to the right. Horizontal lines within boxes represent median values. Black dots represent mean values. Box top and bottom frames represent 25th and 75th percentile. Whiskers represent minimum and maximum values. (C) Illustrates changes in pain ratings over time in FM (left) and HC (right) in response to mid-intensity painful pressure P30. Pain ratings varied significantly depending on whether the P30 pressure was following a red (top row, red color) or a green (bottom row, green color) visual cue (*P* <0.001). Dots (FM) and triangles (HC) represent mean rating values, and error bars represent SDs. FM, fibromyalgia; HC, healthy control; VAS, visual analogue scale.

The following day, the subjects were placed in the scanner for the anatomical scans followed by magnetic resonance spectroscopy (MRS) during rest. The MRS data do not form part of the present article (see https://osf.io/8zqak). Following MRS, the subjects completed an acquisition phase in the fMRI scanner consisting of an identical paradigm of pseudorandomized 10 × P10green and 10 × P50red stimulation on their left calf (Fig. 1A, top row), with pain ratings following each stimulus. Then, subjects had a short break and were instructed to repeat the paradigm (ie, experimental test phase) (Fig. 1A, bottom row). The aim of the test phase was to test the acquired cue-pain associations. During this phase, the initial first 4 stimulations were identical to the preceding run (pseudorandomized 2 × P10green and 2 × P50red) and served as a reminder boost. Following the reminder, both red and green cues were followed by an identical, novel mid-intensity pressure (P30) that the subjects had not previously been exposed to. The mid-intensity pressure, P30, corresponded to each subject's calculated average between P10 and P50 (P30 = ((P50 + P10)/2)) and was presented in a pseudorandomized order of 10 × P30green and 10 × P30red. All stimuli were delivered for 5 seconds and jittered over time with a mean interval between onsets of stimuli of 20 seconds, including 8-second rating time. Both sessions lasted for approx. eleven minutes in total. However, only the results from the test phase were analyzed in the current study.

### 2.3. Questionnaires

The questionnaires were completed during the behavioral testing day, ie, one day before the fMRI session. The Pain Catastrophizing Scale (PCS) is a 13-item scale, each rated on a 5-point scale ranging from 0 = not at all to 4 = all of the time.^40^ The PCS contains 3 subscales: rumination, magnification, and helplessness. Example of items corresponding to the 3 scales in order: “I keep thinking about how much it hurts,” “I wonder whether something serious might happen,” and “I feel I can't stand it anymore.” Higher PCS scores indicate more intense catastrophizing about pain.

Beck's Depression Inventory (BDI) is a 21-item multiple-choice questionnaire assessing the severity of depression.^4^ Scoring allows for the identification of the degree of depressive symptoms, ranging from mild, moderate, to high. Higher BDI scores indicate more severe depressive symptoms.

The State-Trait Anxiety Inventory—State (STAI-S) is a 20-item assessment with 4-point scale (ranging from “almost never” to “almost always”) that is used for measuring state-related anxiety,^6^ ie, current feelings of anxiety. Scores range from 20 to 80, with higher scores indicating higher levels of anxiety (clinical significant cutoff point for STAI-S scale is 39-40). Items include “I feel that difficulties are piling up so that I cannot overcome them” and “I feel like a failure.” In the current study, we chose to correlate pain-related brain activation with STAI-S over the complementary scale State-Trait Anxiety Inventory—Trait. Since, STAI-S measures anxiety in the current moment, which we regard preferably when the scores are used for correlation with fMRI activation. The questionnaires were collected the day before the fMRI scanning session.

In addition, we collected the fibromyalgia impact questionnaire, which is an instrument consisting of 20 items assessing symptoms and disability common to FM. The total score ranges from 0 to 100, where a higher score indicates a lower health status.^5^

### 2.4. Functional magnetic resonance imaging protocol

Magnetic resonance images were acquired with a 3T General Electric 750 MR scanner installed at the MR Research Center, Karolinska Institutet, Stockholm, using an 8-channel head coil. Whole-brain volumes were acquired using a T2*-weighted single-shot gradient echo planar imaging sequence. The following parameters were used: repetition time/ echo time = 2000/30 ms, flip angle = 70°, field of view = 220 × 220 mm, matrix size = 72 × 72, 42 slices, slice thickness = 3 mm with a 0.5-mm gap, acquired through an interleaved slice acquisition mode. Anatomical MR scans were acquired with a high-resolution BRAVO 3D T1-weighted image sequence (1 × 1 × 1-mm voxel size, 176 slices). Anatomical (T2-weighted) scans were investigated by neuroradiologist for clinical abnormalities.

## 3. Statistics

### 3.1. Behavioral data analysis

Clinical characteristics and behavioral data were analyzed in RStudio^37^ (Table 1). Linear mixed-effects model was used to analyze the influence of group, cue color, and time on subjective pain ratings. We used the *lme* function of the *nlme* package^34^ with group (FMS, HC), cue color (red, green) and time as predictor variables, and all interactions. Given that we had multiple ratings from each subject and expect time effects to differ between individuals, we included a random intercept for subjects (assuming baseline variation between individuals) and a random slope (assuming variation in the effects over time). Group differences regarding symptom severity and intragroup analysis of behavioral data were performed using repeated-measures analysis of variance. A *P* < 0.05 was considered statistically significant.

**Table 1:**
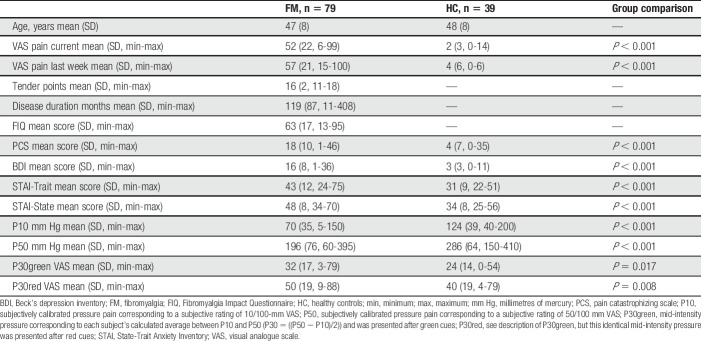
Characteristics of FM subjects and healthy controls.

### 3.2. Functional imaging analysis

Imaging data analyses were performed using the Statistical Parametric Mapping 12 (SPM12) software,^10^ running under Matlab2014 (The MathWorks, Inc, Natick, MA). First, functional and structural images were manually reoriented to the anterior commissure. Second, functional images were spatially realigned using a 6-parameter affine transformation and registered to the mean. Third, individual structural images were coregistered with functional images. Finally, coregistered images were normalized to Montreal Neurological Institute (MNI) space and spatially smoothed using 6-mm full-width half-maximum Gaussian kernel. Frame-wise displacement was used to assess head motion from one volume to the next, converting rotational displacements (sum of the absolute values of the derivatives of the 6 realignment parameters) from degrees to millimeters by calculating displacement on a sphere with a 50-mm radius. In total, 6 FMs and 2 HCs displayed excessive head motion (frame-wise displacement >0.5, in >15% of the images) and were thus excluded from further analyses.

First-level general linear model (GLM) was built on material from the second fMRI session, ie, the testing phase. Regressors of interest were convolved with the canonical hemodynamic response function for cue (green and red) and mid-intensity pressure following green and red cues (P30green, P30red). Button presses and 6 motion parameters were added as regressors of no interest, resulting in a GLM with 11 parameters and one constant. The initial 4 runs consisting of 2 × P50red and 2 × P10green that served as a “reminder boost” were excluded from the GLM.

Second-level one-sample *t* test assessed pain-related brain activity patterns across all participants (FM and HC conjoined) and all P30 pressures regardless of preceding cue. Two-sample *t* test was used to calculate differences in brain activation between groups in response to a medium-intensity pressure (P30) following green and red cues, ie, [P30green] and [P30red], respectively. Paired *t* test was used to calculate within-group differences in brain activation for contrasts [P30green vs P30red]. Three correlational analyses were performed to investigate the relationship between brain activation, pain, and emotional distress (BDI, STAI-S, and PCS) in FM. Because of the low ratings and a lack of variance in affective measures within HCs, no correlational analyses were performed within this group.

Psychophysiological interaction (PPI) task-based functional connectivity was performed to further elucidate and aid the interpretation of the fMRI blood oxygen level dependent analysis finding from the contrast FM [P30green>P30red] including PCS as a covariate of interest. The PPI analyses should be considered secondary. A PPI analysis investigates the interaction between an experimental (psychological) condition and a source region based on a selected volume of interest.^9^ The analysis provides information about the contribution of one region to another in relation to an experimental context, here, during the application of noxious stimulation. Three separate PPI analyses were performed through defining a 4-mm radius around the peak voxels of activation from the contrast FM [P30green>P30red] × PCS, namely, in right thalamus (MNI 16, −30, 6), right dorsal anterior cingulate cortex (dACC) (MNI 4, 26, 30), and left posterior insula (MNI −36, −18, 8). Specifically, we wanted to test whether connectivity from these regions could elucidate whether the activation was pain facilitating (eg, communicating with sensory regions) or pain inhibitory (eg, communicating with pain inhibitory regions such as rostral anterior cingulate cortex, periaqueductal gray, or dorsolateral prefrontal cortex).

For all fMRI analysis, including PPI, statistical significance was considered for cluster-level family-wise error correction for multiple comparisons *P* < 0.05 over the entire brain at an initial statistical threshold of *P* < 0.001 uncorrected with 20 contiguously activated voxels. The anatomical location of cluster peak brain activities is reported in MNI stereotactic atlas coordinates (x, y, z) and labelled through the Automated Anatomical Labelling digital atlas in MRIcron.

## 4. Results

### 4.1. Patient characterization and behavioral results

The descriptive data regarding clinical characteristics as well as group differences are presented in Table 1. As expected, FMSs had increased sensitivity to pressure pain compared with HCs reflected as lower P10 (t(*df*) = 7.36 (69.8); *P* < 0.001) and P50 (t(*df*) = 6.52 (84.74); *P* < 0.001) (Fig. 1B). Both FMSs and HCs rated their experienced pain significantly higher for P30red, compared with P30green (beta = 25.24; t-value = 14.01, *P* < 0.001). Significant interactions were established between group and time (beta = −0.34; t-value = −2.35, *P* = 0.018); time and cue color (beta = −0.58; t-value = −4.16; *P* < 0.001); but not between group and cue color (beta =-3.21; t-value = −1.78; *P* = 0.074) (Fig. 1C). There was no significant three-way interaction (group × time × cue color). To disentangle the directionality of the interactions, post hoc analyses were performed. Post hoc between-group comparisons revealed a significant difference in P30red ratings over time (beta = −0.9; t-value = −2.66; *P* < 0.01), whereas no significant group difference was found in P30green ratings over time (beta = −0.28; t-value = −0.93; *P* > 0.05). Post hoc within-group analysis revealed that HCs adapted their pain ratings over time, ie, increased their ratings for P30green (F(1, 305) = 7.08; *P* <0.01) and decreased their ratings for P30red (F(1,305) = 8.43; *P* < 0.005) (Fig. 1C). Similarly, FMS displayed increased P30green pain ratings over time (F(1,620) = 26.15; *P* < 0.001). However, FMS P30red pain ratings remained stable and elevated throughout the paradigm (F(1,620) = 0.59; *P* >0.05) (Fig. 1C). In FMS, the increased P30green pain ratings were not influenced by PCS ratings (F(1,67) = 0.60; *P* >0.05), BDI ratings (F(1,67) = 2.55; *P* >0.05), or STAI ratings (F(1,67) = 0.69; *P* >0.05). Neither were the elevated and stable FMS P30red ratings influenced by PCS ratings (F(1,67) = 3.52; *P* = 0.065), nor BDI (F(1,67) = 0.84; *P* >0.05), nor STAI (F(1,67) = 0.07; *P* >0.05).

### 4.2. Neuroimaging results

**Figure 2.**
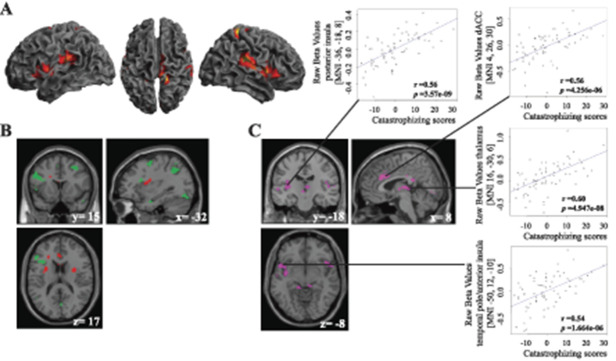
(A) Depicts all participants' brain activation in response to mid-intensity pain pressure (P30), regardless of preceding cue. (B) Depicts brain activation in right anterior insular cortex for FM subjects (green) and HC (red) in response to opposing contrasts. Fibromyalgia subjects (green) revealed greater left anterior insular activation in response to P30 pressure following safety compared with warning cue (ie, FM [P30green>P30red]). Healthy control (red) revealed greater left anterior/mid-insular activation in response to P30 pressure following warning compared with safety cue (HC [P30red>P30green]). (C) Demonstrates increased brain activation in FM subjects (purple) that significantly covaried with higher pain catastrophizing ratings for FM [P30green>P30red] × PCS, namely, in the posterior insula, dorsal anterior cingulate cortex, thalamus, and temporal pole extending into anterior insula. The correlational plots to the right illustrate extracted raw (unscaled) mean beta-weight values from whole clusters of significant brain activation that significantly covaried with higher pain catastrophizing ratings. FM, fibromyalgia; HC, healthy control.

Brain activation in response to P30 pressures (regardless of preceding cue) was associated with significant activation in pain-related regions across all participants, such as primary somatosensory cortex (S1), primary motor cortex (M1), secondary somatosensory cortex (S2), and mid-cingulate cortex (MCC) (Supplementary Table, available at http://links.lww.com/PAIN/B7 and Fig. 2A). No significant group differences were found in cerebral response to applied experimental mid-intensity pressure following red or green cue.

Within-group comparison revealed that HCs displayed significantly greater whole-brain activation when stimulated with mid-intensity pressure following the red compared with the green cue (HC [P30red>P30green]) in a cluster encapsulating left S2 and insular cortex (Table 2 and Fig. 2B). Fibromyalgia subjects, however, revealed a whole-brain pattern of significantly higher brain activity, in the opposite direction (FM [P30green>P30red]) in primary motor cortex (M1) extending to anterior insula; left inferior parietal lobe (IPL), extending to superior parietal lobe; cerebellum; and lingual gyrus (Fig. 2B).

**Table 2:**
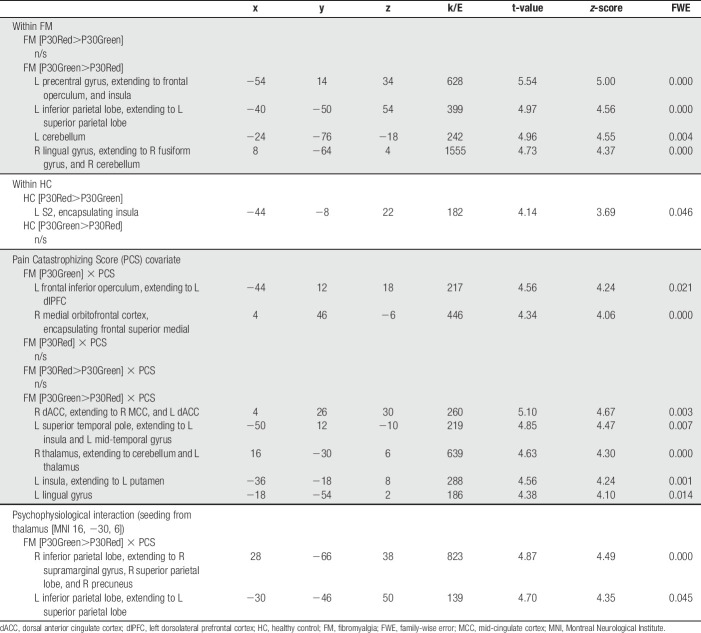
Results from fMRI statistical analyses

Furthermore, brain activation in response to [P30green] and [P30green>P30red] significantly covaried with increased pain catastrophizing scale (PCS) ratings. That is, higher pain catastrophizing scores in FMS covaried with stronger brain activation ([P30green>P30red] × PCS) in bilateral dACC, encapsulating right MCC; left superior temporal pole, encapsulating left anterior insula, and left mid-temporal gyrus; bilateral thalamus, extending to cerebellum; left posterior insula extending to left putamen; and left lingual gyrus (Table 2 and Fig. 2C). Importantly, the PCS correlations did not change when corrected for BDI and STAI-S scores. The BDI and STAI-S scores did not covary with brain activations for these contrasts and neither did PCS, BDI, nor STAI-S scores covary with brain activation for contrast [P3Red] and [P30Red>P30Green] in FMS.

**Figure 3.**
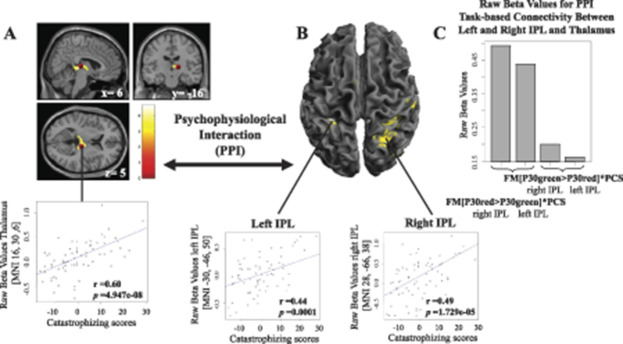
(A) Psychophysiological interaction analysis seeding from right thalamus revealed heightened functional connectivity with (B) bilateral inferior parietal lobes (IPL) that significantly covaried with increased pain catastrophizing ratings in FM subjects when stimulated with identical mid-painful pressure following a green (P30green) or a red (P30red) cue. Specifically, when thalamus was significantly activated during FM [P30green>P30red] × PCS, there was no significant coupling with bilateral IPL. However, when thalamus was not significantly activated during FM [P30red>P30green] × PCS, there was significant positive task-based connectivity between right thalamus and bilateral IPL. (C) Demonstrates raw (unscaled) beta values for FM task-based connectivity (that covaried with pain catastrophizing scores) between right thalamus and right and left IPL. The histogram elucidates that there is not a complete decoupling between thalamus and IPL, but rather significantly less coupling, depending on whether identical mid-painful pressure was following a green or a red cue. All raw beta values were extracted from whole clusters of significant brain activation, not only the peak voxel. FM, fibromyalgia; IPL, inferior parietal lobe.

The PPI analysis, with a seed from the right thalamus (MNI 16, −30, 6) derived from contrast (FM [P30Green>P30Red] × PCS), revealed a significant decrease in functional connectivity with bilateral IPL (Table 2 and Fig. 3) during evoked pain. No significant task-based interaction was found for contrast FM [P30green>P30red] × PCS when seeding from dACC or posterior insula.

## 5. Discussion

In accordance with previous behavioral studies, we report for the first time, neuroimaging data that suggest that FMSs update their cerebral representation to forming new potential pain-related associations while simultaneously maintaining previously formed high-pain associations. Healthy controls adapted all pain-related associations over time. Our results demonstrated that the subjective experience of pain can be modulated through instructed conditioning in both FMSs and HCs, alike. However, pain ratings diverged between groups over time and brain imaging data revealed within-group differences in brain activation. In alignment with our hypothesis, FMSs diverged from HCs, by failing to detect that the painful stimulation following the red cue had been reduced (ie, P50 replaced by P30). Furthermore, FMSs did detect that painful stimulation following the green cue had been increased (ie, P10 replaced by P30), although they did not significantly differ from HCs, contrary to our hypothesis regarding increased attention towards pain-related threats. Nonetheless, FMSs displayed increased brain activation for the identical mid-pressure intensity following green compared with red cue (ie, FM [P30green>P30red]) in a cluster encapsulating primary motor cortex, frontal operculum, and anterior insula; left parietal lobe; cerebellum; and lingual gyrus (Fig. 2B). The cerebral effects were even more pronounced in FMSs when correlated with pain catastrophizing ratings (Fig. 2C), which involves the tendency to magnify the threat associated with pain, ruminating about how much the pain hurts, and feelings of helplessness.^41^ Healthy controls, on the other hand, adapted all pain ratings over time to resemble mid-intensity pain (Fig. 1C) and displayed increased brain activation for the identical mid-intensity pressure following red compared with green cue (ie, the opposite contrast than FMSs: HC [P30red>P30green]) in a cluster encapsulating left S2 and mid-insular cortex (Fig. 2B). No significant group differences were found in cerebral response to P30 following red or green cue.

The behavioral results (collected during fMRI scanning) suggest that HCs did detect the discrepancy between the painful pressure delivered at the acquisition phase and the test phase following both green and red cues (Fig. 1C). Together with the brain imaging data, these results may reflect an adaptive psychological mechanism in HCs, ie, correcting all pain-related associations. By contrast, FMSs only detected the discrepancy between the painful pressure delivered at the acquisition phase and the test phase following green cues, but not following red cues (Fig. 1C). These results suggest that FMSs form new associations linking higher pain to the green cue (ie, P30green), while simultaneously maintaining previous high-pain associations (ie, regarding P30red). Specifically, in combination with increased brain activation for FM [P30green>P30red], the former suggest that FMSs updated the processing and signal value of the painful pressure following the green cue. Whereas, the robustness in ratings and cerebral response to painful pressure following the red cue may be in alignment with reports of dysfunctional extinction behavior in FMS for acquired threat-related associations.^32^ It is tempting to speculate that increased protective responding (eg, failure to extinguish) may be an adaptive process in the short term,^45^ in alignment with a “better-safe-than-sorry” approach to pain. However, in the long run, exaggerated protective behavior may worsen pain disability and contribute to chronic pain maintenance.^44,45^ Moreover, disproportionate responses to nonharmful events and dysfunctional threat identification may lead to increased anxiety as more cues in the environment have the potential to signal harm.^43–45^

To investigate the potential effects of emotional distress, we assessed the relationship between FMS ratings of anxiety, depression, and pain catastrophizing and cerebral pain processing in FMS. Although anxiety and depression are common comorbidities in FM,^28^ we specifically found that pain catastrophizing scale (PCS) (but neither anxiety nor depression) were associated with neural responses to mid-intensity pressure following the green cue (FM [P30Green] × PCS). The effects were even more pronounced for mid-intensity pressure following green compared with red cue (FM [P30Green>P30Red] × PCS). Importantly, neither contemporary use of antidepressant nor anticonvulsant medication was allowed in the current study, and the correlational effects of pain catastrophizing remained stable even when controlling for depression (ie, BDI) and anxiety (ie, STAI-S). Conversely, pain catastrophizing did neither covary with brain activation for mid-intensity pressure following red cue (FM [P30red] × PCS), nor red compared with green cue (FM [P30red>P30green] × PCS).

Higher FM PCS ratings covaried with cerebral activation for FM [P30green] × PCS in left frontal inferior operculum, encapsulating left dorsolateral prefrontal cortex (dlPFC), and in medial orbitofrontal cortex (OFC)/medial prefrontal cortex (mPFC). The dlPFC is a region eminently implicated in top-down attentional processes,^39^ and cognitive reappraisal of pain in FMSs,^20^ while the medial OFC process behavioral and emotional salience.^36^ Speculatively, the increased dlPFC and OFC/mPFC activation in high pain catastrophizing FMSs may reflect increased attention and salience toward the slightly higher painful pressure following the green cue in the test phase. Likewise, it has been suggested that the relationship between catastrophizing and heightened pain experience may be mediated through attentional processes,^41^ which may explain why we observed neural correlations with PCS, but not between PCS and pain ratings per se. Furthermore, neuroimaging meta-analysis of pain catastrophizing^11^ and instructed fear conditioning^29^ report overlapping coactivation of the same regions that we observed in our whole-brain analysis to covary with increased PCS ratings in FMSs when stimulated with an identical mid-intensity painful pressure following green compared with red cue (ie, FM [P30green>P30red] × PCS) (Fig. 2C), namely, in dACC/MCC, insula, bilateral thalamus, mPFC, putamen, superior, and middle parts of the temporal lobe.^29^ Worthy of note, these brain regions are also commonly associated with pain processing.^22^ However, our whole-brain analysis did not reveal any differential brain activation in sensory regions such as S1 or S2 (possibly due to the identical mid-intensity pressure input delivered following both green and red cues). Taken together, these results suggest that higher pain catastrophizing ratings in our FM subjects may be related to increased neural activation in regions related to pain processing, salience detection, fear, and top-down attentional processes when stimulated with an identical mid-intensity painful pressure following green compared with red cue.

Last, to aid in the interpretation whether the pain catastrophizing-related increases in neural activation were related to pain-facilitating or pain-inhibitory effects, we conducted a task-based connectivity analysis seeding from thalamus (Fig. 3A). Our results revealed a functional dissociation between thalamus and bilateral IPL that covaried with increased FM pain catastrophizing ratings, during identical mid-intensity pressure stimulation depending on whether the pressure was following a red or a green cue (FM [P30green>P30red] × PCS, or FM [P30red>P30green] × PCS) (Figs. 3B and C). Specifically, when thalamic activation was significantly increased (during FM [P30green>P30red] × PCS), there was no significant task-based connectivity with IPL. Whereas, when thalamus decreased in activation (during the opposite contrast, ie, FM [P30red>P30green] × PCS), its connectivity to bilateral IPL significantly increased. The observed decrease in functional connectivity may suggest that thalamus exert regulatory effects on IPL in high pain catastrophizing FMS for the identical input pain pressure depending on whether it followed a red or a green cue. The parietal cortex plays a key role in pain perception through its involvement in sensorimotor integration and supporting body awareness.^3,12^ Atrophy to the parietal cortex in neurodegenerative diseases is associated with interoceptive impairments,^13^ and a recent meta-analysis on neuroimaging studies reveal that thalamus, together with IPL, are among the most likely clusters of activation when investigating interoception.^1^ In FM, low interoceptive accuracy is associated with increased symptom severity,^7^ and higher disruption of external signals.^42^ In line with the fear-avoidance model,^43,45^ Zaman et al.^48^ proposed that associative fear learning impairs the ability to discriminate between different bodily sensations, reducing interoception and contributing to more intense and frequent pain experiences.^48^ Speculatively, in the current study, the observed disrupted thalamic IPL functional connectivity may reflect a sensory disintegration that is more pronounced among high pain catastrophizing FMS, which may lead to a tendency to overestimate incoming sensory signals and/or excessively shut off incoming sensory signals.

### 5.1. Limitations

We chose to establish pain-cue association awareness through verbal instruction during acquisition and by using colored cues with an intrinsic signal value, which may have interfered with extinction. This decision was based on previous reports of dysfunctional noninstructed contingency learning in FMS^15,31,44^ and a brain-imaging meta-analysis.^29^ Also, to keep the procedure as simple as possible, no self-report measures of CS expectancy and fear were included. Our goal was to achieve stable fMRI results during the test phase to study how the FM and HC brain process novel pain pressure stimulation following pain conditioning. Our results may not be applicable to noninstructed contingency learning in everyday life.

## 6. Conclusion

The current study provides, to the extent of our knowledge, the first brain imaging data to support previous behavioral studies,^15,31,32^ suggesting that FMS preferentially form new potential pain-related associations while simultaneously maintaining previous high-pain associations that are not as painful anymore. Healthy control, however, adapted all pain-related associations over time. The effect was even more pronounced among high pain catastrophizing FMS. Increased responses to pain-related threats in FMS may contribute to dysfunctional pain-protective behaviors and disability^44^ and should be addressed when treating FM.

## Conflict of interest statement

The authors have no conflicts of interest to declare.

## Appendix A. Supplemental digital content

Supplemental digital content associated with this article can be found online at http://links.lww.com/PAIN/B7.
